# Triborheological Study under Physiological Conditions of PVA Hydrogel/HA Lubricant as Synthetic System for Soft Tissue Replacement

**DOI:** 10.3390/polym13050746

**Published:** 2021-02-28

**Authors:** Laura C. Duque-Ossa, Gustavo Ruiz-Pulido, Dora I. Medina

**Affiliations:** Tecnologico de Monterrey, School of Engineering and Science, Atizapan de Zaragoza, Estado de Mexico 52926, Mexico; A01747663@itesm.mx (L.C.D.-O.); A01166117@itesm.mx (G.R.-P.)

**Keywords:** triborheology, polyvinyl alcohol hydrogel, hyaluronic acid lubricant, wear, coefficient of friction

## Abstract

In soft tissue replacement, hydrophilic, flexible, and biocompatible materials are used to reduce wear and coefficient of friction. This study aims to develop and evaluate a solid/liquid triborheological system, polyvinyl alcohol (PVA)/hyaluronic acid (HA), to mimic conditions in human synovial joints. Hydrogel specimens prepared via the freeze–thawing technique from a 10% (*w*/*v*) PVA aqueous solution were cut into disc shapes (5 ± 0.5 mm thickness). Compression tests of PVA hydrogels presented a Young’s modulus of 2.26 ± 0.52 MPa. Friction tests were performed on a Discovery Hybrid Rheometer DHR-3 under physiological conditions using 4 mg/mL HA solution as lubricant at 37 °C. Contact force was applied between 1 and 20 N, highlighting a coefficient of friction change of 0.11 to 0.31 between lubricated and dry states at 3 N load (angular velocity: 40 rad/s). Thermal behavior was evaluated by differential scanning calorimetry (DSC) in the range of 25–250 °C (5 °C/min rate), showing an endothermic behavior with a melting temperature (Tm) around 231.15 °C. Scanning Electron Microscopy (SEM) tests showed a microporous network that enhanced water content absorption to 82.99 ± 1.5%. Hydrogel achieved solid/liquid lubrication, exhibiting a trapped lubricant pool that supported loads, keeping low coefficient of friction during lubricated tests. In dry tests, interstitial water evaporates continuously without countering sliding movement friction.

## 1. Impact Statement

Musculoskeletal diseases represent one of the main causes of labor and academic absence in the world, so they represent a public health problem that causes economic, time, and production losses [1]. Osteoarthritis is the most common degenerative disease associated with synovial joints, cartilage and bone wear, bone volume loss and deterioration, osteophyte formation, and inflammation [2,3]. Around the world, it is present in more than 25% of the adult population [2]. However, most treatments are ineffective over the long term, and thus, cartilage replacement surgery is commonly used for treating the disease. The procedure involves the use of synthetic elements which emulate soft tissues and synovial joint behavior. In order to determine components that can be used for treatments, this study proposes the use of biocompatible triborheological systems under physiological conditions, operating with synthetic materials with non-isotropic surfaces in terms of roughness.

## 2. Introduction

The human body suffers degradation and loss of motor skills over time, affecting quality of life in relation to the mental and physical health of individuals in areas of functional independence [4,5]. The joint cavity is composed by cartilaginous tissue, synovial fluid, subchondral bone, and sometimes menisci [3]. The synovial fluid (SF) is a biological lubricant composed by mucopolysaccharides, proteins, and lipids, which form a protective lubricant film for articular joints by reducing friction and wear [6,7,8]. The articular cartilage (AC) creates a fibrous connection between 2 or more bones [9,10]. It is integrated by chondrocytes and an extracellular matrix (ECM), which give strength and help to generate good mechanical properties against stress loads [11,12].

**Figure 1:**
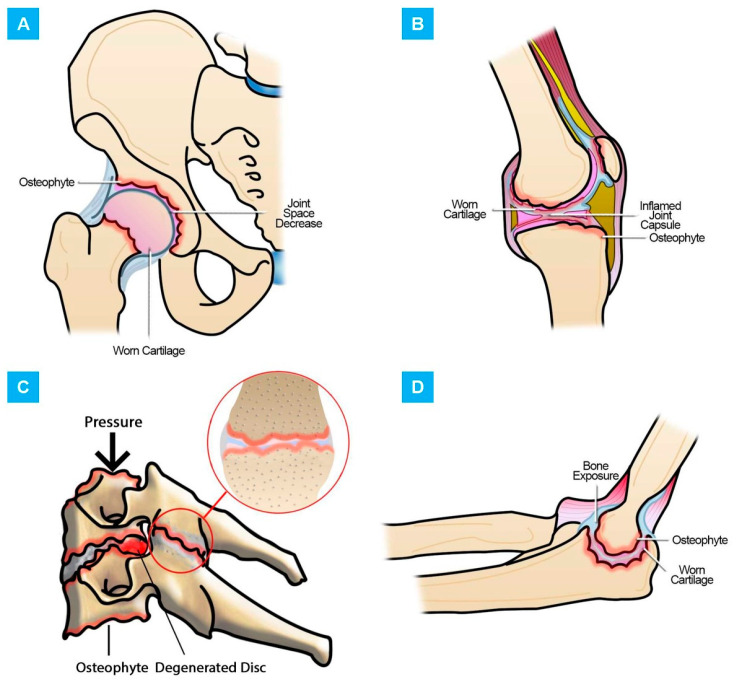
Damage explanation in the joints: (**A**) Hip osteoarthritis (OA); (**B**) knee OA; (**C**) facet OA; (**D**) elbow OA.

When an articulation begins its failure stage, it means that its AC has lost its properties, decreasing its volume and deteriorating [10,13,14]. This leads to the appearance of diseases such as osteoarthritis (OA) [15,16]. OA is a degenerative disease associated with wear and tear of joints such as the knee, hip, foot, hand, spine, and elbow, among others (Figure 1), presenting loss of AC, osteophyte formation, inflammation, alteration of SF composition, subchondral bone sclerosis, and varying degrees of synovitis [2,3,17,18]. Worldwide, it is present in more than 25% of the population over 18 years of age and is responsible for an economic cost of around 89.1 billion USD per year worldwide. [2,17]. OA pathology is derived not only from age, but from lifestyle, weight, overuse, injury, and genetic and gender conditions [2,19,20]. For example, cervical facet joint OA affects older adults, increasing its incidence with age [21,22]. Its prevalence is around 54–67% of spinal pain symptoms [23,24]. For hip OA, the population over 85 years old presents a risk of around 25% of suffering from the disease. [25]. In the case of knees, it is estimated that 6% of adults worldwide have signs of OA [26].

Medical and engineering researchers have tried to reduce the progress of OA and correct its effects with pharmacological interventions, physical therapy, cell therapy, and tissue engineering through surgical methods [3,27,28]. AC as an avascular tissue does not regenerate easily, so surgical methods are most commonly used for restoring or replacing cartilage surfaces [10,29]. Tissue engineering has generated advances by repairing cartilage and developing implants for surgery, considering human body mechanics and its response to external agents [30,31]. Cell culture techniques, clinical trials, scaffolds, and synthetic materials are also used [30,32].

The use of biomaterials has focused on polymers, mainly hydrogels [10]. They have hydrophilic structures (high water content), softness, flexibility, and biocompatibility [33,34,35]. Synthetic hydrogels can mimic AC behavior in terms of lubrication and mechanical performance. They can even improve upon AC’s toughness and strength, providing extraordinary mechanical properties [36,37,38]. Within the polymer group, polyvinyl alcohol (PVA) is the most relevant in current studies [39,40,41]. PVA has been used to improve and to regenerate different tissues and organs in the human body, such as arterial phantoms, corneal implants, and cartilage tissue replacements [41]. Specifically, for AC substitution, several sliding studies have demonstrated its performance under diverse temperatures, charges, and velocity conditions. Thus, PVA is considered an excellent choice in tissue engineering for friction reduction and low wear results [42,43,44].

For tests, the use of lubricants to minimize damage in artificial cartilage is imperative. Hyaluronic acid (HA), a component of synovial fluid, is used as an alternative to increase joint lubrication due to its viscoelastic properties. [45,46,47]. This viscous fluid provides protection against articular wear [48,49]. Today, HA is commonly used in tribological tests that involve the study of PVA hydrogels [50].

Due to the above, the goal of this research was to evaluate the potential of PVA hydrogels for soft tissue replacement, such as AC, using HA as lubricant, under physiological conditions of sample temperature (37 °C) and using a saline solution as a solvent for sample preparation to recreate fluid conditions in the human body. Additionally, pressures applied during the tests were equivalent to the impact upon joints on a daily basis.

## 3. Materials and Methods

### 3.1. Preparation of PVA Hydrogels

The specimens were produced from a 10% PVA aqueous solution (Sigma Aldrich, St. Louis, MO, USA, 13 × 10^4^ Daltons, 99% hydrolyzed, 10% *w*/*w*) in distilled water by the freeze–thawing technique (FT) [51]. The mixture was stirred continuously at 80 °C for 3 h using a magnetic stirrer to achieve complete homogenization. The solution was placed in glass petri dishes and four FT cycles were performed. The freezing stage was carried out for 12 h at −20 °C, while the thawing stage was carried out at room temperature for 12 h.

For triborheological and mechanical tests, FT hydrogel samples were cut in a disc shape with 5 ± 0.5 mm thickness and 45 mm diameter.

HA lubricant solution was prepared using 4 mg of HA powder (Lifecore Biomedical, Chaska, MN, USA, 8 × 10^5^ Daltons) per mL of saline solution (0.15 M NaCl) under stirring conditions of 150 rpm for 6 h.

### 3.2. Equilibrium Water Content Measurements

For equilibrium water content measurement (EWCM), the procedure proposed by Sardinha et al. in 2013 was followed. PVA hydrogels samples were cut into 25 × 25 mm squares with 5 ± 0.5 mm thickness, weighted, and submerged in distilled water at ambient temperature until they reached a stable weight. Samples were removed from water and weighted again. Weight difference between the hydrated hydrogel (*Wh*) and the dehydrated hydrogel (*Wd*) gives the EWCM in percentage, using Equation (1) [44].

(1) $$EWCM\;\left(\%\right)=\;\frac{Wh-Wd}{Wh}\times 100$$

### 3.3. Mechanical Tests

Compression tests were carried out, emulating joint cavity conditions in a Shidmadzu UH universal testing machine (Shimadzu Corporation, Kyoto, Japan): humidity, preheating at 37 °C, compression speed of 1 mm/min with a maximum load of 4500 N. Considering Equations (2)–(4), and values obtained from stress vs. strain curves, the parameters were calculated as described by Gupta et al. [52,53]:

(2) $$\sigma =\;\frac{F}{A}$$

(3) $$\varepsilon =\;\frac{\delta }{L_{0}}=\;\frac{L_{0}-L_{f}}{L_{0}}$$

(4) $$E=\;\frac{\sigma }{\varepsilon }$$

where *σ* is the stress, *F* is the applied load, *A* is the area where the load is applied, *ε* is the strain, *δ* is the calibrated length, *L*_0_ is the initial length, *L_f_* is the final length, and *E* is the modulus of elasticity or Young’s modulus [52].

### 3.4. Rheological and Friction Tests

**Figure 2:**
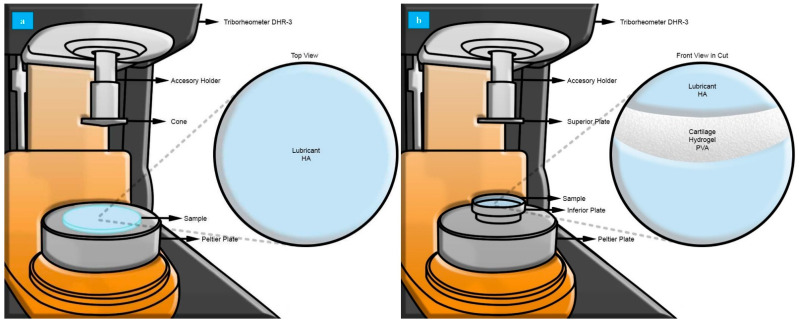
Characterization in a Discovery Hybrid Rheometer DHR-3. (**a**) Rheological study using cone-and-plate stainless steel configuration with an angle of 0.09969°, a diameter of 60 mm, and a truncation gap of 23 um. Samples used were 4 mg/mL hyaluronic acid (HA) solution. (**b**) Triborheological study using plate-and-plate stainless steel configuration with a diameter of 40 mm. Samples used were 10% polyvinyl alcohol (PVA) hydrogel with 5.0 ± 0.5 mm thickness and 45 mm diameter, hydrated with 4 mg/mL HA solution.

Tests were performed with a Discovery Hybrid Rheometer DHR-3 (TA Instruments, New Castle, DE, USA, maximum charge 50 N) using cone-and-plate (0.9969°) configuration for rheological characterization (Figure 2a) and plate-and-plate configuration for the triborheological part (Figure 2b).

All the experiments were performed at 37 °C to mimic the physiological conditions of the human body. HA in 4 mg/mL concentration in saline solution 0.10 M NaCl was used as lubricant fluid [54]. Samples were submerged at all times in lubricant to ensure their complete hydration. Velocity was fixed between 0.1 and 100 rad/s, and contact force was applied in a range of 1–20 N. The coefficient of friction changes were continuously monitored during tests.

Rheology tests were carried out in 3 steps: conditioning sample (37 °C during 10 s), frequency sweep (0.01–100 Hz, 10% strain), and flow sweep (shear rate 0.001–100 s^−1^). Triborheological tests were conducted in 2 steps: conditioning options active (37 °C for all charges: 1 N, 3 N, 5 N, 10 N, 15 N, 20 N) and flow sweep (angular velocity 0.1–100 rad/s).

### 3.5. Surface Analysis

The morphology of the PVA hydrogel and identification of wear mechanism traces was observed by JEOLJSM-6360 scanning electron microscopy (SEM, JEOL USA, Peabody, MA, United States) at 20 kV. Samples were managed using high vacuum at level 3 of the beam diameter, working with secondary electrons at magnifications between 50 and 1000×. Samples were cut into squares of 10 × 10 mm (thickness of 5 ± 0.5 mm) and gold-sputtered to enhance conductivity. Samples were analyzed in surface distribution, porosity, and wear tracks before and after triborheological tests.

### 3.6. Thermal Behavior

The thermal properties of dried hydrogels were measured by differential scanning calorimetry (DSC, Q10 TA Instruments, United States). Dried hydrogels (2–5 mg) were placed in an aluminum pan and heated at a rate of 5 °C/min from 25–250 °C in a nitrogen atmosphere. The melting enthalpy, Δ*H_m_*, was determined by integrating the area under the melting peak over the range 208–242 °C. The crystallinity (*Xc*) was calculated by

(5) $$X_{c}=\frac{\Delta H_{m}}{\Delta H_{c}}\times 100\%$$

where Δ*H_c_* = 138.6 J/g, which represents the heat required for melting a 100% crystalline PVA sample [55].

## 4. Results and Discussion

### 4.1. Surface Morphology and Water Content

Naturally, AC has a high water content to mitigate the stress generated from contact forces applied during the movement of synovial joints. Hydrogels must present similar swelling properties to correctly mimic the natural AC performance in terms of super water-absorbing capability, elasticity, compressive mechanical properties, wear reduction, and low coefficient of friction during sliding [44,56,57]. For that reason, the water content absorption of PVA hydrogels was analyzed by measuring the equilibrium water content in percentage (EWCM %). Results showed an average EWCM of 82.99 ± 1.5% in accordance with Table 1.

The percentage of water stored in the hydrogel is not dependent on the initial weight of the sample. Even thickness variation does not affect the swelling range, which is maintained around the same value. The swelling behavior of hydrogels is a consequence of the diffusion of water molecules inside the polymer network [58], which depends mainly on the sample’s porosity and porous size, which were similar in all the samples as all were prepared under the same conditions [57]. Therefore, the obtained swelling values for the material are adequate for its purpose, because the water content of a natural CA is around 80% [59,60].

**Figure 3:**
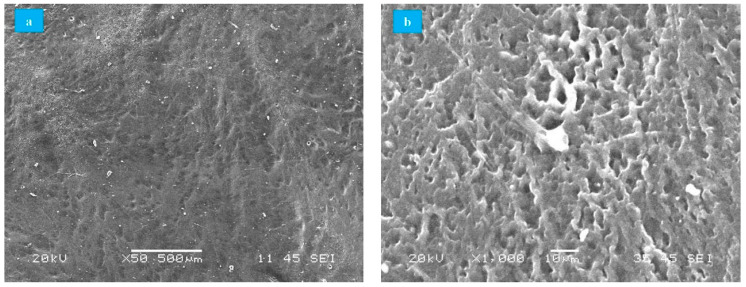
SEM of PVA hydrogels. (**a**) Surface distribution, (**b**) porosity (High vacuum, 50–1000×, secondary electrons).

Unworn surfaces of PVA hydrogel were visualized in SEM equipment for surface distribution and porosity evaluations. According to Figure 3a, the surface was smooth and largely homogeneous despite the presence of inclusions adhered from the environment (dust particles) that adhered during the process of dehydration. Moreover, SEM micrographs of PVA hydrogels showed the porous network that results from the spaces generated by the ice crystals formed during the FT process [56]. Those pores allowed water absorption, reducing hydrogel wear (Figure 3b). Similar results have been reported by Sardinha et al. and Li et al. in studies of PVA hydrogels under similar conditions [43,44].

### 4.2. Mechanical Tests

**Figure 4:**
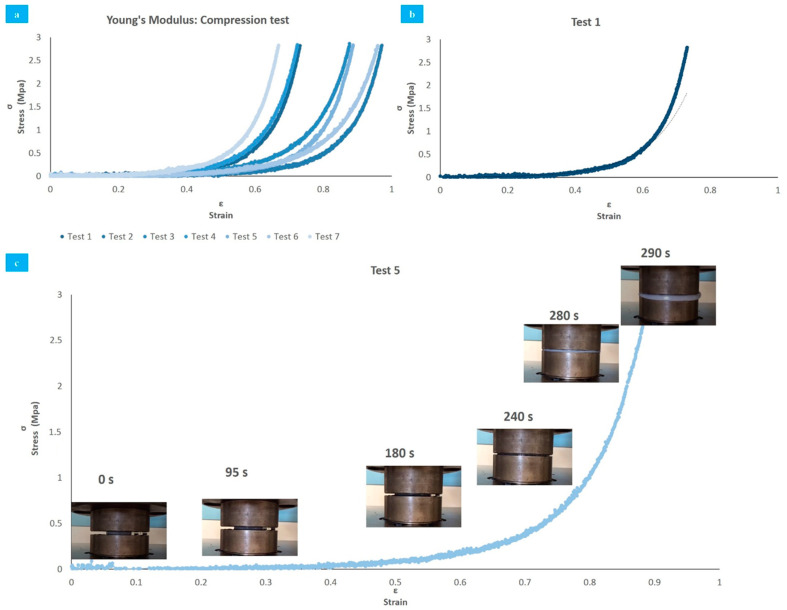
Compression tests (Up to 4500 N, compression rate 1 mm/min). (**a**) Linear regression of all tests; (**b**) exponential behavior of compression modulus in test 1; (**c**) test 5 deformation over time.

The stress–strain curves obtained from 7 tests of compression after 4 FT cycles are reported in Figure 4a. All samples exhibited a non-linear behavior and were plotted from the data obtained by the instrument. Using a linear regression, the average Young’s modulus obtained was 2.26 ± 0.52 MPa. The displacement observed was caused by the standard deviation present in the initial thickness of the samples.

In accordance with the curves, the behavior of the hydrogel samples accurately fits an exponential relationship. For example, test 1 exhibited a correlation coefficient R^2^ higher than 0.9, indicating a positive proportionality of the effort against deformation, which explains the viscoelastic characteristics of the material (flexibility and rapid response to external loads) [61].

Figure 4b shows that when the deformation rises from 33.7% to 70%, Young’s modulus increments from 0.05 MPa to 2.7 MPa, which represents an increase of 51 times. This mechanical behavior is expected, and comparable to natural AC. When the cartilage is subjected to a considerable high load activity (such as running or jumping), the compression module increases to resist high stress, reducing deformation and preventing cartilage damage [61].

Measurement of Young’s modulus exhibited similar results to previous studies that involved mechanical characteristics of natural and synthetic cartilages. The viscoelastic behavior of the samples during the tests is shown in Figure 4c, through images that reveal the deformation (sample 5) at certain times.

### 4.3. Rheological and Friction Tests

**Figure 5:**
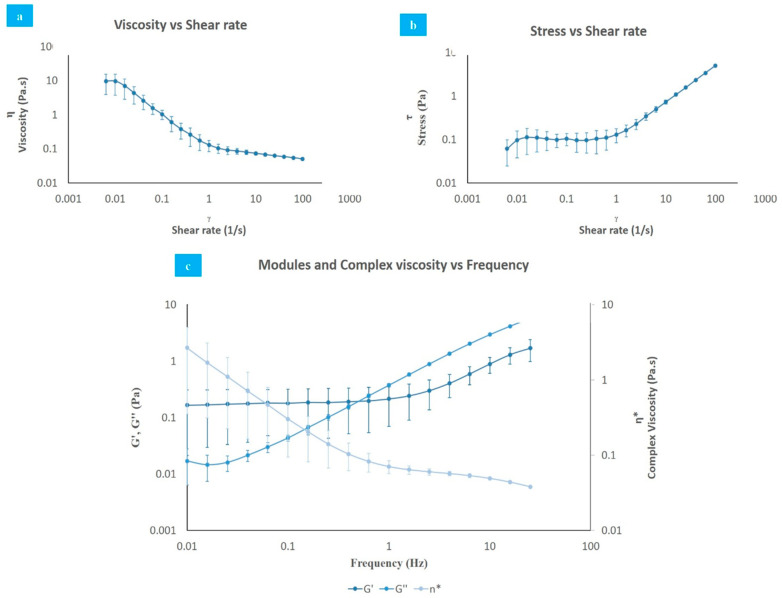
Rheological test. (**a**) Viscosity vs. shear rate; (**b**) shear stress vs. shear rate; (**c**) modules and complex viscosity vs. frequency (humidity, 37 °C, 0.01–100 Hz, 0.001–100 s^−^^1^).

Rheological test data are displayed in Figure 5. HA presented a non-Newtonian behavior, where the viscosity decreases (Figure 5a) as the shear stress increases (Figure 5b), in a clear example of shear thinning [62].

The evaluation of complex viscosity (η*), elastic (G′), and viscous (G″) modulus (Figure 5c) showed that at low frequencies, the sample exhibited an elastic behavior (G′ > G″), while at high frequencies, it presented a viscous behavior (G″ > G′). This behavior is typical of substances in solution, known as “weak gels”, and is contrary to the results of pure HA, which initially behaves viscously (G″ > G′) [54,63,64,65]. Additionally, the number of FT cycles should increase the elastic modulus, which is related to the gel strength and porosity, because the cycles stimulate the formation of crystalline junction zones among PVA chains and the appearance of hydrogen bonds resulting in PVA densification. As more couplings and bonds are formed, the sol–gel transition occurs and the sample no longer flows as a polymer solution, and its viscoelastic properties exhibit a solid-like behavior as its macromolecules lose their identity and become part of a large three-dimensional network, expanding throughout the entire sample volume [58].

The crossover frequency from a viscous to an elastic state demonstrates the viscoelastic behavior expected from a lubricant. The changing behavior of the modulus indicates an increased deformation of the cartilage at high frequencies, enhancing its resistant properties [66].

**Figure 6:**
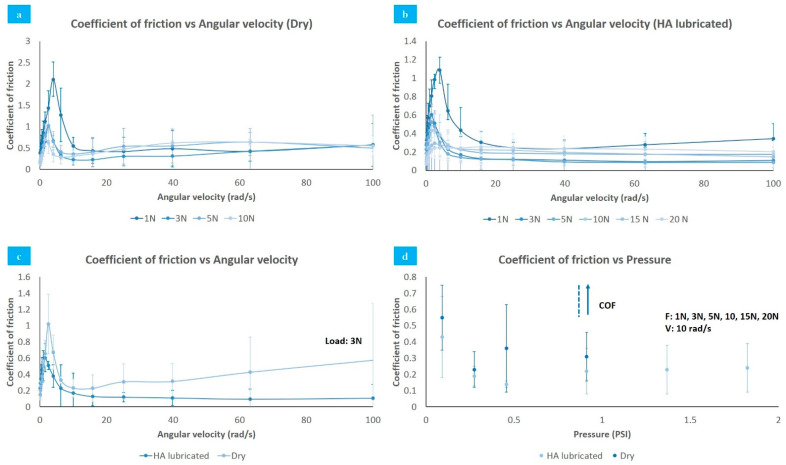
Coefficient of friction vs. angular velocity. (**a**) Dry; (**b**) HA lubricated; (**c**) coefficient of friction vs. angular velocity: Lubrication comparative; (**d**) coefficient of friction vs/pressure (0.1–100 rad/s, 1–20 N).

Data obtained of the coefficient of friction of PVA synthetic cartilage against stainless steel plates under different factors are shown in Figure 6. Figure 6a,b indicates that coefficient of friction is affected by angular velocity, increasing between 0 and 4 rad/s, followed by a quick decrease among 4–10 rad/s. Finally, it rises slowly again after 10 rad/s. The speed generates the same effect on the sample, regardless of whether it is lubricated or not. Specifically, coefficient of friction increased from 0.19 to 0.3 when the velocity incremented from 0 to 4 rad/s and decreased from 0.3 to 0.14 when velocity moved from 4–10 rad/s under 5 N load.

As seen in Figure 6c, lubrication causes a decrease in the coefficient of friction. For a 3 N load and an angular velocity of 40 rad/s, it goes from 0.11 to 0.31 when switching from HA lubrication to a dry state. In a dry state, the large amount of water accumulated by the samples contributes to counteracting the sliding movement, while in a lubricated state, the transition of the lubricant from elastic to a viscous state minimizes the friction generated at high angular velocity values.

Pressure also generates an effect upon coefficient of friction. For 0.46 PSI with HA lubricant, the coefficient of friction was 0.14 at an angular velocity of 10 rad/s. For the same conditions, the coefficient of friction increased to 0.24 under a 1.82 PSI load as Figure 6d shows. Meanwhile, in dry samples, the coefficient of friction increased exponentially after 0.91 PSI.

Under an applied load, the coefficient of friction decreases, followed by an increase as the water or HA (depending on the conditions of the experiment) is squeezed out from the PVA hydrogel through its porous network. As the interstitial fluid expands from the porous structure to the conjunction and suffers instantaneous pressurization supporting a fraction of the applied load, it produces a coefficient of friction reduction.

**Figure 7:**
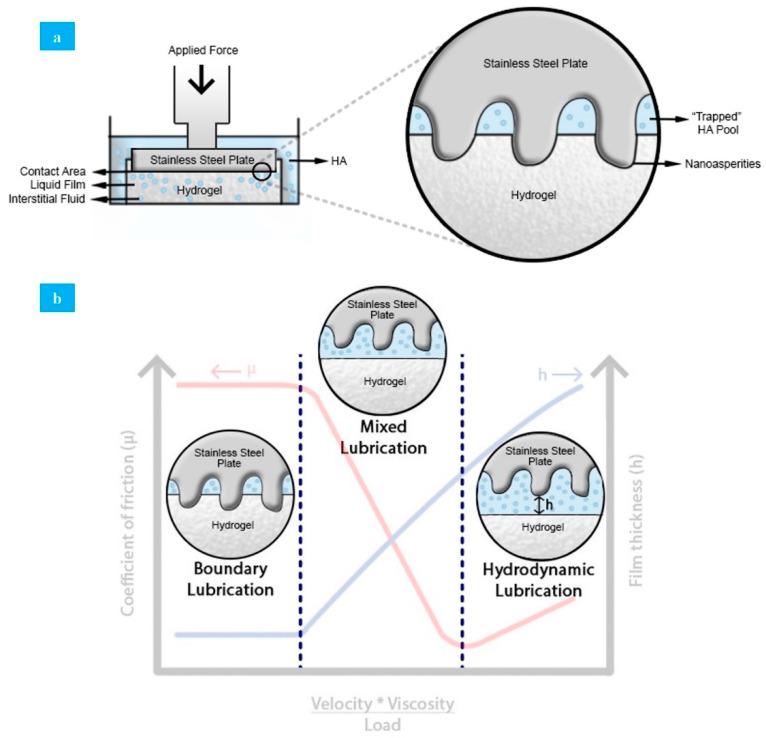
(**a**) Lubrication mechanism in PVA hydrogel under applied force in solid–solid contact. (**b**) Lubrication mechanisms applied to a PVA hydrogel/HA lubricant system.

This behavior could be explained by comparing 3 parts of the lubrication mechanisms of natural articular cartilage. Boundary lubrication is an important lubrication mechanism in natural synovial joints, forming a molecular film [45]. This film takes up the boundary lubricants (HA, lubricin, among others), providing a defense against contact [67]. PVA hydrogels do not present a molecular layer that would recreate boundary lubrication similar to synovial joints, but the initial administration of lubricant prior to each test mimics this function to an extent. HA, at the beginning of the test, forms a small film that avoids hard contact between PVA hydrogel and stainless-steel plate, diminishing surface wear. This film is provided by a lubricant pool (trapped lubricant) that gets out from the hydrogel, creating an interface between the steel and the hydrogel that reduces the effective contact area between surfaces, and consequently the coefficient of friction (Figure 7a) [68]. Moreover, the liquid layer could be continuously fed by the trapped and external lubricant through hydrogel swelling to maintain its pressurization and to form new layers during sliding [69]. Interstitial fluid rehydration is limited by hydrogel swelling capacity.

After a while, during contact between both surfaces, only a small portion of the rough parts (nano asperities) of the stainless-steel plate interact directly, producing friction. This is a consequence of the presence of mixed lubrication, where the HA supports a fraction of the load. However, continuous increase in the load causes the displacement of the lubricant from the hydrogel surface, enlarging the contact area, which leads to coefficient of friction increase [70]. This behavior is known as hydrodynamic lubrication. In this case, high pressurization of the interstitial fluids occurs under applied normal loads as the fluid supports most of the loads transmitted through articular surfaces [71]. As such, friction force in the contact surface is reduced considerably, ensuring that coefficient of friction maintains low values. By contrast, under dry sliding conditions, the liquid inside the porous structure expands as lubricant during the initial period. Afterward, the superficial liquid evaporates without replacing, which produces an increase in the coefficient of friction and surface wear. In natural articular cartilage, mixed and hydrodynamic behavior are explained by solid/liquid theory that considers the collagen–proteoglycan network as a solid phase interacting with interstitial liquid and ions as a fluidic phase [72]. In Figure 7b, all mechanisms applied to the solid/liquid system form by PVA hydrogel and HA as a lubricant can be observed.

In general, all results reported are comparable to previous multifactor studies. Pan et al. and Shi et al. showed that an increment in the coefficient of friction is related to the increasing load, as well as with the effect of lubrication or dryness of the sample [72,73]. Likewise, values reported for coefficient of friction in natural AC and synthetic PVA AC are similar. According to Kobayashi et al., natural AC has a coefficient of around 0.1 and synthetic PVA cartilage made by FT cycles between 0.7 and 0.8 [74]. Thus, PVA hydrogel shows a higher coefficient of friction than natural articular cartilage.

### 4.4. Thermal Behavior

**Figure 8:**
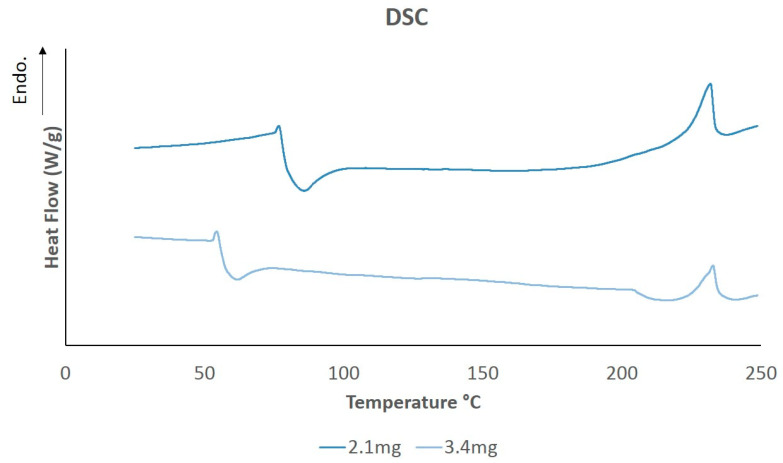
Differential scanning calorimetry (DSC) thermal diagram of PVA samples at different weights.

The DSC melting curves of dried PVA hydrogels of different weights are shown in Figure 8. Peaks reported at around 232.31 °C represent the approximate melting temperature, Tm, of the samples. The values obtained are related to sample crystallinity, as has been expressed in previous studies [55]. The melting enthalpy and crystallinity percentages of PVA hydrogel samples were calculated by analyzing the area under the curve from the melting peaks using Universal Analysis 2000 (TA Instruments) software, as shown in Table 2.

In general, PVA hydrogels prepared by the FT technique exhibited similar crystallinity degrees to values reported in literature [75]. Crystallinity can be affected by factors like FT cycles, concentration of aqueous solution, and molecular weight of PVA [55,76].

## 5. Conclusions

The mechanical and triborheological properties of PVA synthetic cartilage/HA lubricant were investigated, and they demonstrated attributes for the generation of potential prototypes for AC replacement by testing, under physiological conditions, the effects of swelling, load, angular velocity, and lubrication. Surface morphologies of the samples were analyzed with SEM and mechanical properties were determined by a universal machine. PVA showed an average EWCM of 82.99 ± 1.5%, which is comparable to natural AC’s EWCM of 80%. Additionally, the compression Young’s modulus was higher than 2, providing good resistance to stress by reducing deformation and preventing cartilage damage. In rheology studies, the crossover frequency influenced the behavior of the lubricant, changing its nature from elastic state to a more viscous form and indicating a good performance.

It was also confirmed, through triborheological tests, that the coefficient of friction exhibited values between 0.1 and 0.4, which are comparable with those of the coefficient of natural AC that is around 0.1. Therefore, 10% PVA hydrogel lubricated with 4 mg/mL HA could represent a great mechanical alternative for future advances in replacing damaged AC in human joints under moderate load.

## Figures and Tables

**Table 1:** Water content measurements of dry and hydrated samples at different weights.

Test	*W_h_* (g)	*W_s_* (g)	EWCM (%)
1	1.48	0.23	84.46
2	1.72	0.26	84.88
3	1.73	0.26	84.97
4	1.91	0.31	83.77
5	2.45	0.42	82.86
6	2.54	0.44	82.68
7	2.70	0.49	81.85
8	3.11	0.59	81.03
9	3.32	0.50	84.94
10	3.38	0.64	81.07
11	3.45	0.67	80.58
12	3.62	0.56	84.53
13	3.72	0.63	83.06
14	4.32	0.79	81.50
15	4.73	0.82	82.66
Average		82.99	
Standard Deviation		1.50	

**Table 2:** DSC results for melting temperature and crystallinity. Measurements were made on a Q10 TA Instruments. Samples were heated at a rate of 5 °C/min from 25–250 °C in a nitrogen atmosphere.

Sample Weight (mg)	T_m_ (°C)	ΔH_m_ (J/g)	X_c_ (%)
2.1	231.83	13.92	10.04
3.4	232.79	9.78	7.06

## Data Availability

The data presented in this study are available on request from the corresponding author.
